# Supplementary material for: Molecular Epidemiology of Rotavirus A in Calves: Evolutionary Analysis of a Bovine G8P[11] Strain and Spatio-Temporal Dynamics of G6 Lineages in the Americas
Source: Viruses. 2023 Oct 19;15(10):2115. doi: 10.3390/v15102115 (PMC10611311; doi:10.3390/v15102115)
Supplement: Supplementary file 1 [file viruses-15-02115-s001.zip › Supplementary materials 22-9-23 (Louge et al., 2023).docx]

Table S1. Beef and dairy farms and fecal samples collected from neonatal calves from 2007 to 2010.

| Farm acronym | Year of sampling | Production system | District | Production Region | Feeding | Rearing system | Energy/protein concentrate | Sampled calves | Diarrehic  calves | Non-diarrheic  calves | Vaccination of dams |
| --- | --- | --- | --- | --- | --- | --- | --- | --- | --- | --- | --- |
| EA | 2007 | Beef | Pila | Cuenca del Salado | Nursed by cows | Seeded/native pastures | NA^*^ | 16 | 8 | 8 | Yes |
| SA | 2007 | Beef | General Paz | Cuenca del Salado | Nursed by cows | Seeded/native pastures | NA | 26 | 6 | 20 | Yes |
| LD | 2007 | Beef | Rauch | Cuenca del Salado | Nursed by cows | Seeded/native pastures | NA | 7 | 2 | 5 | Yes |
| CR | 2008 | Beef | Mar Chiquita | Cuenca del Salado | Nursed by cows | Seeded/native pastures | NA | 13 | 10 | 3 | No |
| DD | 2008 | Beef | Azul | Cuenca del Salado | Nursed by cows | Seeded/native pastures | NA | 10 | 5 | 5 | Yes |
| LA | 2008 | Beef | Olavarria | Depresion de Laprida | Nursed by cows | Seeded/native pastures | NA | 7 | 6 | 1 | No |
| LB | 2008 | Beef | Mar Chiquita | Cuenca del Salado | Nursed by cows | Seeded/native pastures | NA | 28 | 20 | 8 | Yes |
| LR | 2008 | Beef | Mar Chiquita | Cuenca del Salado | Nursed by cows | Seeded/native pastures | NA | 7 | 4 | 3 | Yes |
| ED | 2008 | Beef | Pila | Cuenca del Salado | Nursed by cows | Seeded/native pastures | NA | 24 | 16 | 8 | Yes |
| SJR | 2008 | Beef | General Pueyrredon^&^ | Cuenca del Salado | Nursed by cows | Seeded/native pastures | NA | 9 | 6 | 3 | Yes |
| EB | 2009 | Beef | Gonzales Chaves | Depresion de Laprida | Nursed by cows | Seeded/native pastures | NA | 12 | 8 | 4 | Yes |
| G | 2009 | Beef | Gonzales Chaves | Depresion de Laprida | Nursed by cows | Seeded/native pastures | NA | 8 | 4 | 4 | Yes |
| LM | 2009 | Beef | Olavarria | Depresion de Laprida | Nursed by cows | Seeded/native pastures | NA | 3 | 3 | 0 | Yes |
| LC | 2010 | Beef | Olavarria | Depresion de Laprida | Nursed by cows | Seeded/native pastures | NA | 12 | 6 | 6 | Yes |
| VT | 2008 | Dairy | Mar Chiquita | Cuenca Mar y Sierras | Milk replacer | Stakes | Yes | 22 | 10 | 12 | Yes |
| LE | 2008 | Dairy | Loberia | Cuenca Mar y Sierras | Milk | Stakes | No | 13 | 7 | 6 | Yes |
| EC | 2008 | Dairy | General Pueyrredon | Cuenca Mar y Sierras | Milk replacer | Stakes | Yes | 11 | 4 | 7 | Yes |
| LSyLM | 2008 | Dairy | Lincoln | Cuenca Oeste | Milk | Stakes | Yes | 14 | 11 | 3 | Yes |
| VA | 2008 | Dairy | Tandil | Cuenca Mar y Sierras | Milk + milk replacer | Stakes | Yes | 15 | 7 | 8 | Yes |
| SC | 2008 | Dairy | General Pueyrredon | Cuenca Mar y Sierras | Milk replacer | Stakes/hutches | Yes | 18 | 7 | 11 | No |
| PF | 2008 | Dairy | Loberia | Cuenca Mar y Sierras | Milk + milk replacer | Stakes | No | 6 | 5 | 1 | No |
| LM | 2008 | Dairy | Balcarce | Cuenca Mar y Sierras | Milk | Stakes | Yes | 16 | 9 | 7 | No |
| LA | 2008 | Dairy | General Pueyrredon | Cuenca Mar y Sierras | Whey + milk | Stakes | Yes | 15 | 7 | 8 | Yes |
| CS | 2008 | Dairy | Mar Chiquita | Cuenca Mar y Sierras | Milk replacer | Stakes | Yes | 15 | 3 | 12 | Yes |
| LE | 2009 | Dairy | Pehuajo | Cuenca Oeste | Milk | Stakes | Yes | 18 | 14 | 4 | Yes |
| DM | 2009 | Dairy | Balcarce | Cuenca Mar y Sierras | Milk | Stakes/hutches | Yes | 16 | 12 | 4 | No |
| DC | 2009 | Dairy | Olavarria | Cuenca Mar y Sierras | Milk | Stakes | Yes | 10 | 7 | 3 | No |
| SP | 2009 | Dairy | Olavarria | Cuenca Mar y Sierras | Milk + milk replacer | Stakes | Yes | 10 | 2 | 8 | No |
| DA | 2009 | Dairy | Olavarria | Cuenca Mar y Sierras | Milk | Stakes | Yes | 6 | 5 | 1 | No |
| DR | 2009 | Dairy | Rivadavia | Cuenca Oeste | Milk | Stakes | Yes | 19 | 17 | 2 | Yes |
| TSIL | 2010 | Dairy | Carmen de Areco | Abasto Norte | Milk | Hutches | Yes | 13 | 7 | 6 | Yes |
| AG | 2010 | Dairy | Tres Arroyos | Cuenca Mar y Sierras | Milk | Stakes | No | 3 | 3 | 0 | No |

^&^District just outside the beef production region of “Cuenca del Salado”; * Not applicable (NA)

| Viral Protein  (segment) | Primers (sense) | (Degenerate) Sequences (5̕- 3̕)^a^ | Positions | Purposes | RT conditions  (ºC/min) | PCR conditions  (ºC/min) | | | | Product length (bp) | Genotypes and G6 lineages | Ref. |
| --- | --- | --- | --- | --- | --- | --- | --- | --- | --- | --- | --- | --- |
|  |  |  |  |  | Denaturation,  cDNA synthesis,  inactivation | Initial  denaturation  step | Cycle No. | Denaturation, annealing, elongation | Final extension  step |  |  |  |
| VP7 (9) | Gra-5 (+) | GGC TTT AAA AGC GAG AAT TT | 1-20 | RT-PCR | (94/5), (37/60), (70/15) | (94/3) | 35 | (94/1),(44/1), (72/2) | (72/7) | 1062 |  | [11, 44] |
|  | Gra-3 (-) | GGT CAC ATC ATA CAA CTC TA | 1062-1044 | RT-PCR |  |  |  |  |  |  |  |  |
|  | Gra-5 (+) | GGC TTT AAA AGC GAG AAT TT | 1-20 | SnM |  | (94/2) | 30 | (94/1), (44/1), (72/2) | (72/7) |  |  | [11] |
|  | N167 (-) | TAT AAG CAG AAA GCT GTA A | 168-150 | SnM |  |  |  |  |  | 168 | G6(IV) |  |
|  | H167 (-) | AAC GAA TAA AAA TCT GTA A | 168-150 | SnM |  |  |  |  |  | 168 | G6(III) |  |
|  | H500 (-) | TCC AAT TCC AAC GTT GAA A | 500-482 | SnM |  |  |  |  |  | 168-500 | G6(III) |  |
|  | HT8 (-) | CGG TTC CGG ATT AGA CAC | 273-256 | SnM |  |  |  |  |  | 273 | G8 | [11, 44] |
|  | ET10 (-) | TTC AGC CGT TGC GAC TTC | 714-697 | SnM |  |  |  |  |  | 714 | G10 |  |
| VP8* (4) | Con3 (+) | TGG CTT CGC TCA TTT ATA GAC A | 11-32 | RT-PCR | (94/5), (37/60), (95/5) | (94/3) | 35 | (94/1), (47/1), (72/2) | (72/7) | 877 |  | [11, 44] |
|  | Con2 (-) | ATT TCG GAC CAT TTA TAA CC | 887-868 | RT-PCR |  |  |  |  |  |  |  |  |
|  | Con2 (-) | ATT TCG GAC CAT TTA TAA CC | 887-868 | SnM |  | (94/2) | 35 | (94/0.5), (47/1), (72/0.75) | (72/7) |  |  |  |
|  | P1 K (+) | ACC AAC GAA CGC GGG GGT G | 264-284 | SnM |  |  |  |  |  | 624 | P[1] |  |
|  | P5 K (+) | (R)CC AGG TGT C(R)C ATC AGA G | 336-354 | SnM |  |  |  |  |  | 552 | P[5] |  |
|  | pB223(+) | GGA ACG TAT TCT AAT CCG GTG | 574-594 | SnM |  |  |  |  |  | 314 | P[11] |  |

Table S2. Primers and cycling conditions used for the G and P typing of bovine RVA field strains.

^a^ Degenerate primer; R = A/G.


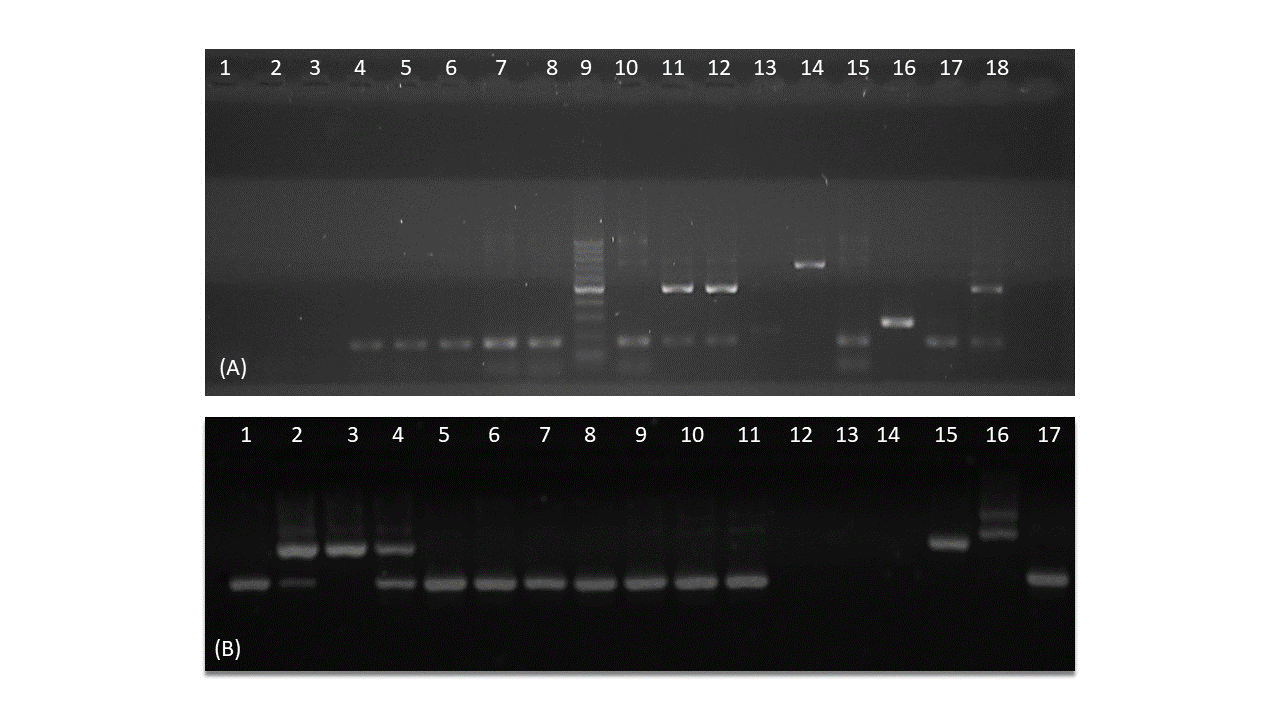


Supplementary Figure S1. G and P genotyping of RVA field strains. (A) Representative G genotypes and G6 lineages determined by SnM RT-PCR assay and visualized by agarose gel electrophoresis (1.8%). Lanes 1-3: RT-PCR mix, SnM PCR mix, and negative control sample; lanes 4-8: samples 6-LB, 12-LB, 3-SJR, 8-EB, and 15-G [G6(IV), 168 bp] from beef calves; lane 9: bp marker (100-1000 bp); lanes 10-12: 5595-DR [G6(IV), 168 bp], 9-DM and 7765-LE [G6(III), 168 and 500 bp] from dairy calves; lane 13: negative sample; lanes 14-18: strain B223 (G10, 714 bp), strain Indiana from a challenged calf [G6(IV)P[5], 168 bp], strain NCDV Cody I-801 (G8P[1], 273 bp), strain Indiana [G6(IV)P[5], 168 bp], reference field strain [G6(III)P[11], 168 and 500 bp]. (B) Representative P genotypes determined by SnM RT-PCR assay and visualized by agarose gel electrophoresis (1.8%). Lanes 1 and 5-11: B-CS, C-LM, 10-SC, 14-SC, 16-SC, 9-DM, A-LE, 7765-LE (P[11], 314 bp) from dairy calves; lanes 2 and 4: 171-LA and 3-PF (P[5]+P[11], 552 and 314 bp) mixed P genotypes from dairy calves; lane 3: 173-LA (P[5], 552 bp) from a dairy calf; lanes 12-14: RT-PCR mix, SnM PCR mix, and negative control sample; lanes 15-17: strain Indiana (G6(IV)P[5], 552 pb), strain NCDV Cody I-801 (G8P[1], 624 pb), strain B223 (G10P[11], 314 pb).


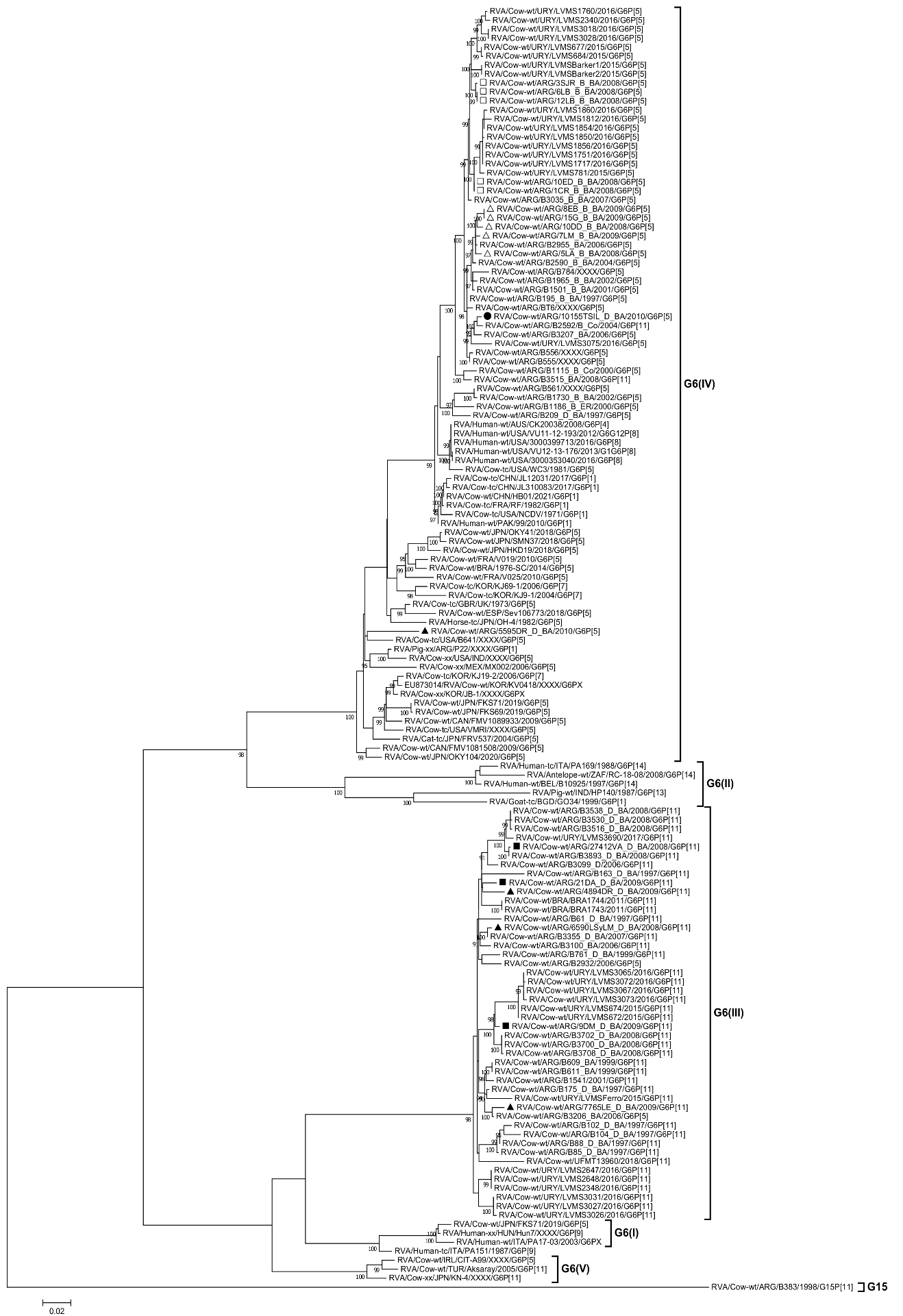


Supplementary Figure S2. Phylogenetic tree of VP7-G6 genotype. The phylogram was reconstructed using the nucleotide substitution model HKY+F+I+G4 and the maximum likelihood method, as implemented in IQ-TREE v1.6.12. The strain RVA/Cow-wt/ARG/B383/ 1998/G15P[11] was included as outgroup. Similar VP7 sequences retrieved from GenBank as well as sequences of RVA strains from G6(I) to G6(V) lineages were included in the analysis. Ultrafast bootstrap values (10,000 replicates) ≥90% are shown as branch nodes. In Argentinean strains: the name, type of production system, and province of collection are indicated when available. Abbreviations: D/B, Dairy or Beef; BA, Buenos Aires; Co, Cordoba; ER, Entre Rios. Beef regions: □ “Cuenca del Salado”; “Depresion de Laprida”. Dairy regions: ■ “Mar y Sierras”; ▲ “Oeste”; ● “Abasto Norte”.


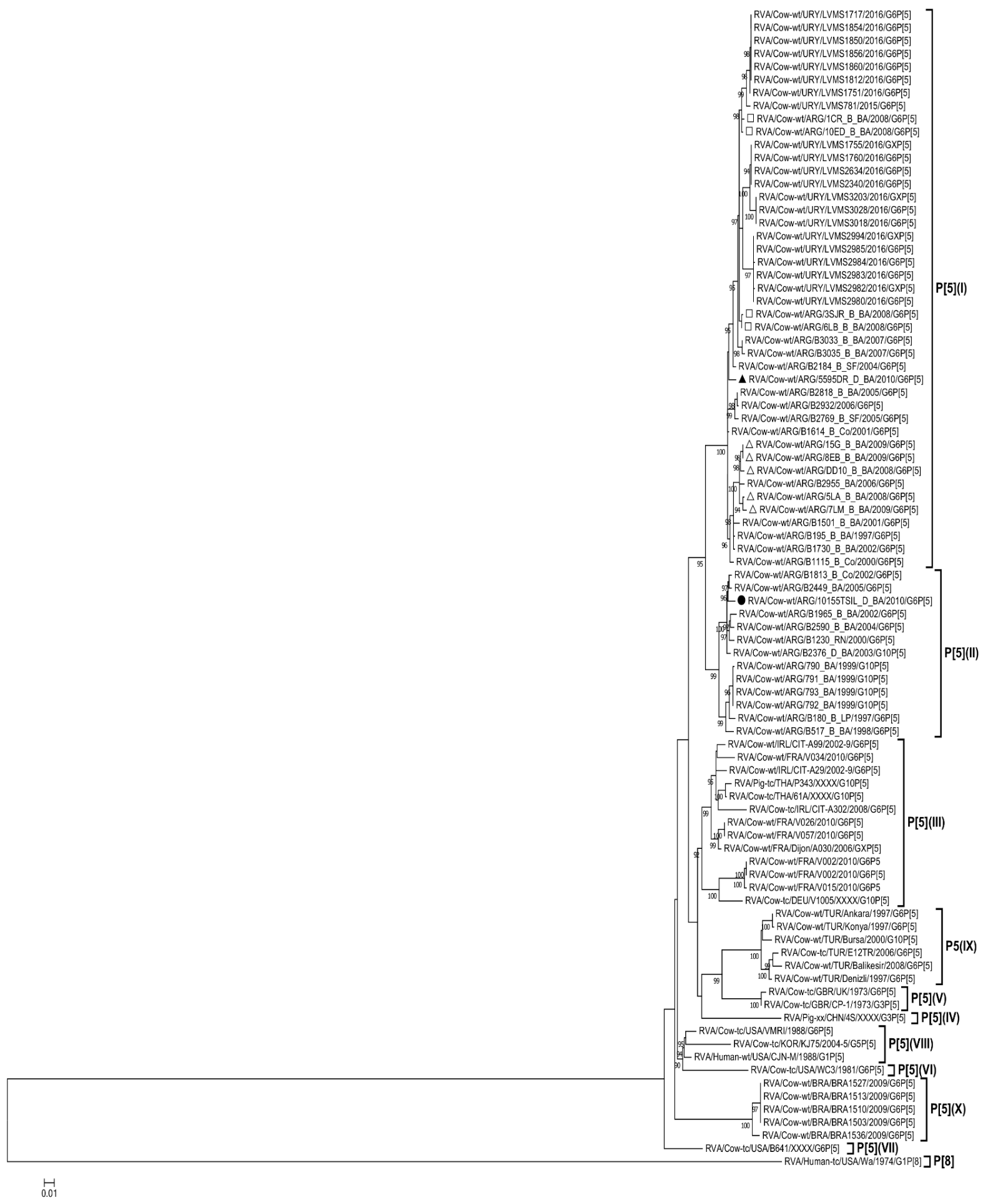


Supplementary Figure S3. Phylogenetic tree of VP8*-P[5] genotype. The phylogram was reconstructed using the nucleotide substitution model TN+F+G4 and the maximum likelihood method, as implemented in IQ-TREE v1.6.12. The strain RVA/Human-tc/USA/Wa/1974/G1P[8] was included as outgroup. Similar VP8* sequences retrieved from GenBank as well as sequences of RVA strains from P[5](I) to P[5](X) lineages were included in the analysis. Ultrafast bootstrap values (10,000 replicates) ≥90% are shown as branch nodes. In Argentinean strains: the name, type of production system, and province of collection are indicated when available. Abbreviations: D/B, Dairy or Beef; BA, Buenos Aires; Co, Cordoba; LP, La Pampa; SF, Santa Fe; RN, Rio Negro. Beef regions: □ “Cuenca del Salado”; “Depresion de Laprida”. Dairy regions: ▲ “Oeste”; ● “Abasto Norte”.


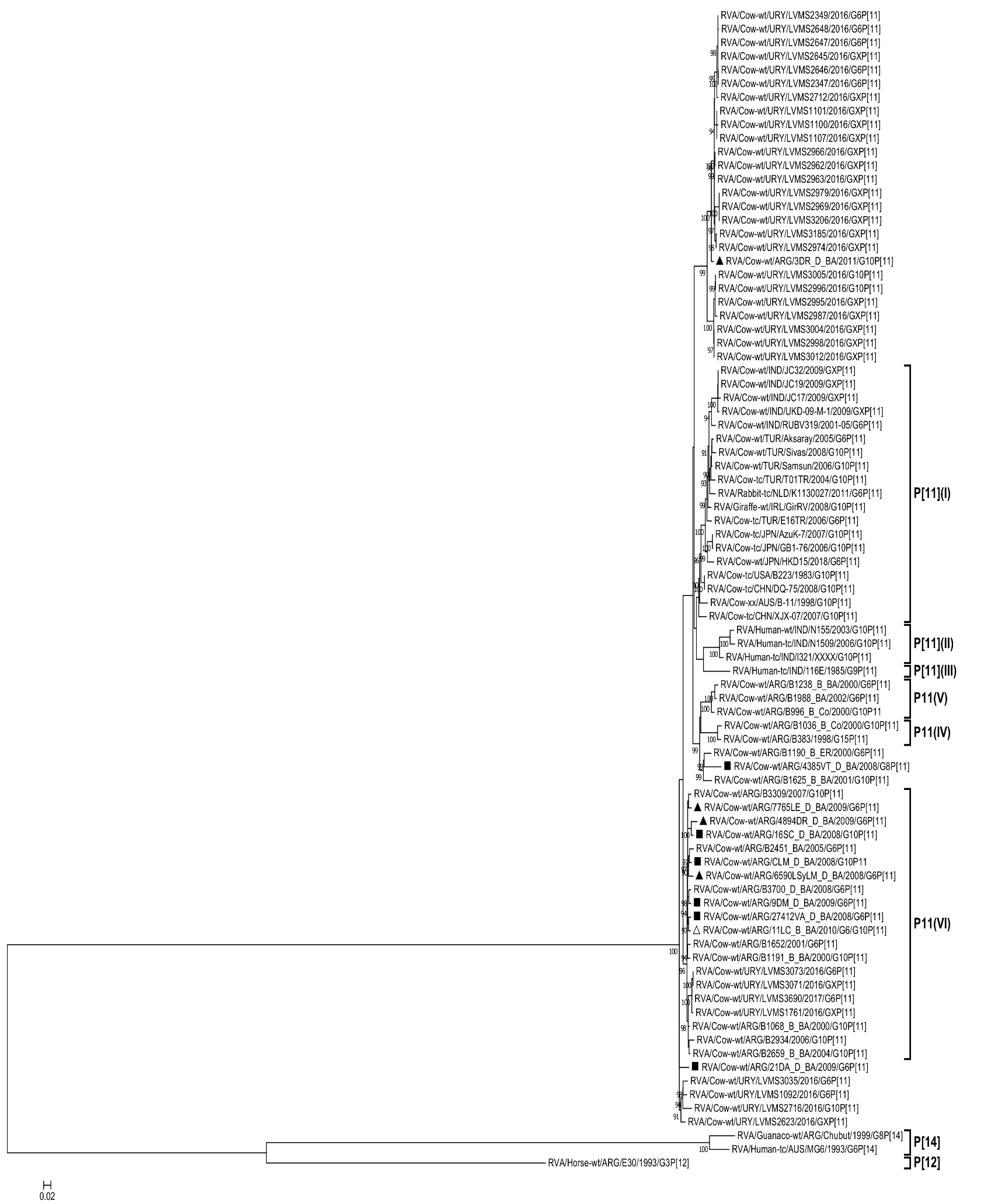


Supplementary Figure S4. Phylogenetic tree of VP8*-P[11] genotype. The phylogram was reconstructed using the nucleotide substitution model TPM2+F+G4 and the maximum likelihood method, as implemented in IQ-TREE v1.6.12. The strains RVA/Guanaco-wt/ARG/Chubut/1999/G8P[14], RVA/Human-tc/AUS/MG6/1993/G6P[14], and RVA/Horse-wt/ARG/E30/1993/G3 P[12] were included as outgroups. Similar VP8* sequences retrieved from GenBank as well as sequences of RVA strains from P11[I] to P11[VI] lineages were included in the analysis. Ultrafast bootstrap values (10,000 replicates) ≥90% are shown as branch nodes. In Argentinean strains: the name, type of production system, and province of collection are indicated when available. Abbreviations: D/B, Dairy or Beef; BA, Buenos Aires; Co, Cordoba; ER, Entre Rios. Beef regions: □ “Cuenca del Salado”; “Depresion de Laprida”. Dairy regions: ■ “Mar y Sierras”; ▲ “Oeste”.


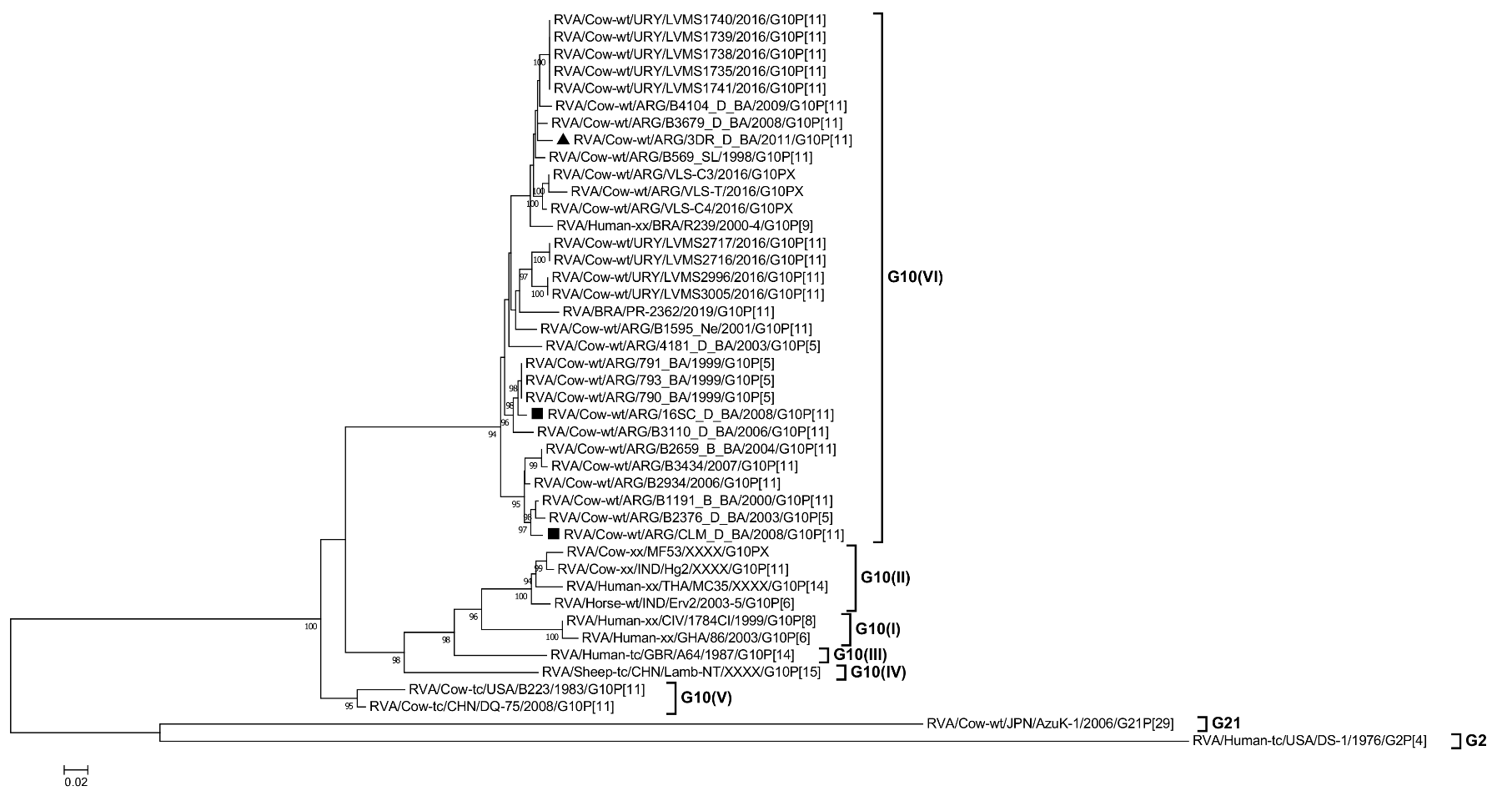


Supplementary Figure S5. Phylogenetic tree of VP7-G10 genotype. The phylogram was reconstructed using the nucleotide substitution model TPM3+F+G4 and the maximum likelihood method, as implemented in IQ-TREE v1.6.12. The strains RVA/Cow-wt/JPN/AzuK-1/2006/G21P[29] and RVA/Human-tc/USA/DS-1/1976/G2P[4] were included as outgroups. Similar VP7 sequences retrieved from GenBank as well as sequences of RVA strains from G10(I) to G10(VI) lineages were included in the analysis. Ultrafast bootstrap values (10,000 replicates) ≥90% are shown as branch nodes. In Argentinean strains: the name, type of production system, and province of collection are indicated when available. Abbreviations: D/B, Dairy or Beef; BA, Buenos Aires; Ne, Neuquen; SL, San Luis. Dairy regions: ■ “Mar y Sierras”; ▲ “Oeste”.


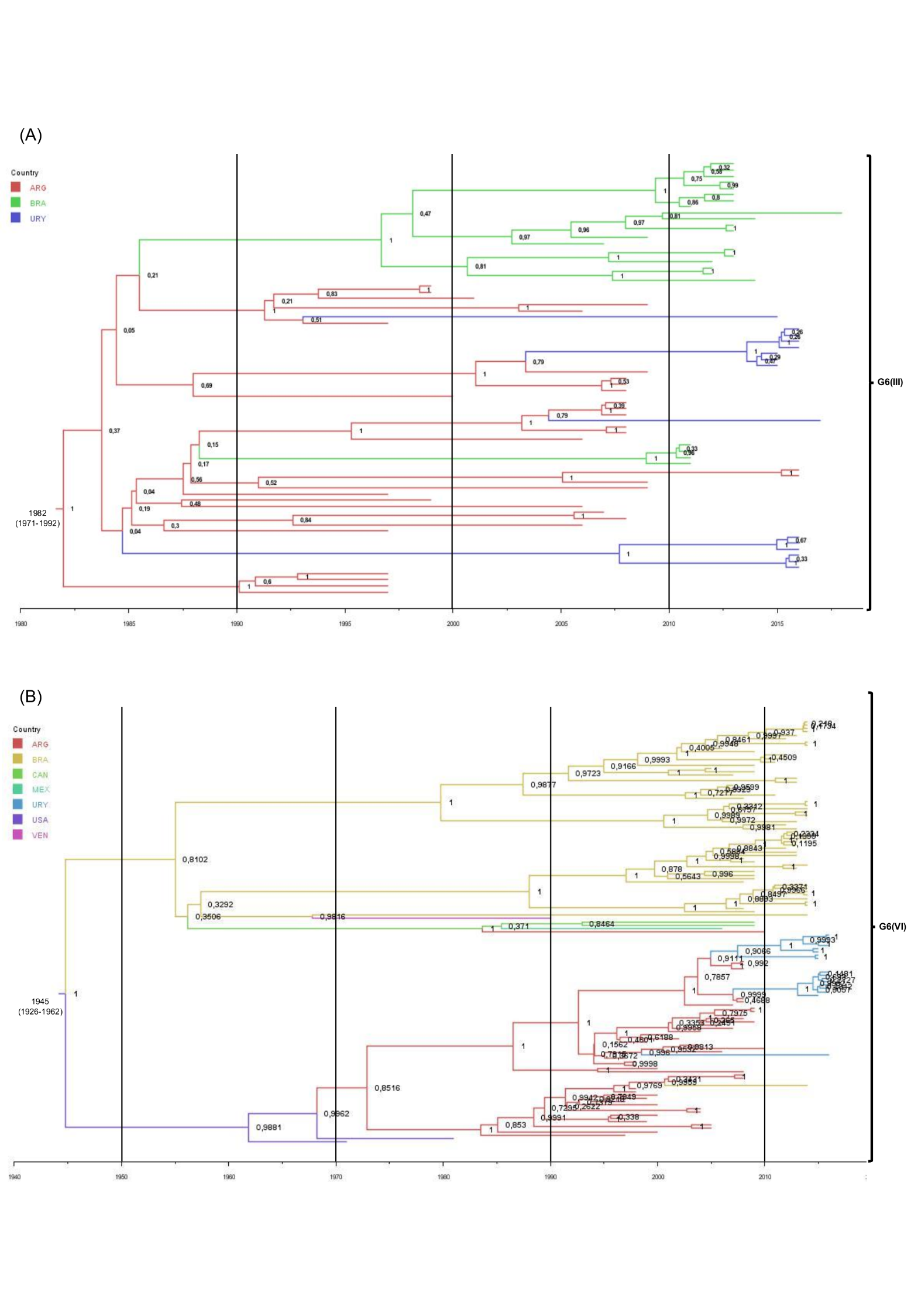


Supplementary Figure S6. Maximum clade credibility trees (MCCT) of bovine RVA strains corresponding to G6 lineage III [G6(III)] (A) and G6 lineage IV [G6(IV)] (B) in American countries. The color of the branches represents the most probable country of origin. Posterior probability values are shown. Time of the most recent common ancestor (TMRCA) and the 95% highest posterior density (95% HPD) intervals are indicated below the tree nodes. Country abbreviations using the three-letter code (alpha-3) (ISO 3166): ARG, Argentina; BRA, Brazil; CAN, Canada; MEX, Mexico; URY, Uruguay; USA, the United States of America; VEN, Venezuela.


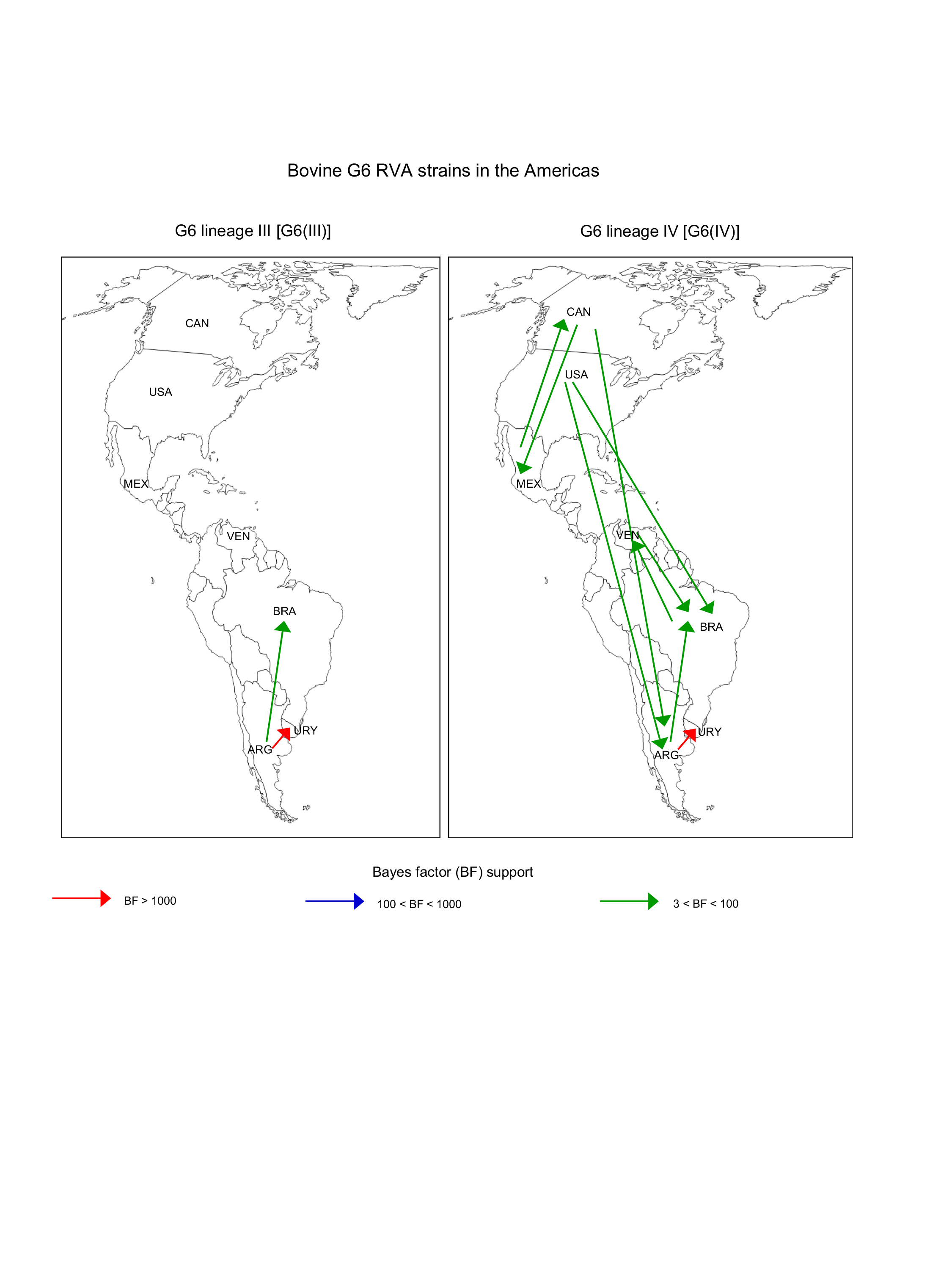


Supplementary Figure S7. Phylogeographic reconstruction of G6 lineage III [G6(III)] and G6 lineage IV [G6(IV)] among RVA strains circulating in American countries. Line color represents the relative strength of connection between countries according to the Bayes Factor (BF) test: red arrows, decisive support with BF > 1000; blue arrows, strong support with 100 < BF < 1000; and green arrows, supported rates with 3 < BF <100. Country abbreviations using the three-letter code (alpha-3) (ISO 3166): ARG, Argentina; BRA, Brazil; CAN, Canada; MEX, Mexico; URY, Uruguay; USA, the United States of America; VEN, Venezuela. (B) Inferred spread pattern of G6(III) and G6(IV) lineage strains in American countries.

**Pipeline for phylodynamic analyses**

To ensure that our datasets contained sufficient information for reliable phylodynamic and phylogeographic studies, we followed the checkpoint pipeline stated by Mavian et al., 2020, which consists in assessing:

**1) Phylogenetic signal:**

All the three possible unrooted trees for a set of four sequences (quartets), which are selected randomly from the dataset, are reconstructed by maximum likelihood approach using the selected substitution model. Then the posterior probability of each tree is plotted on a triangular surface. Fully resolved trees fall at the corners and the unresolved quartets in the center of the triangle (Schmidt et al., 2002). Simulation studies have shown that for sequences to be considered robust in terms of the phylogenetic signal, the center area of the likelihood mapping must include <33 dots (Strimmer and von Haeseler, 1997).

The phylogenetic noise in the analyzed datasets was low to rather low (see the following figure).


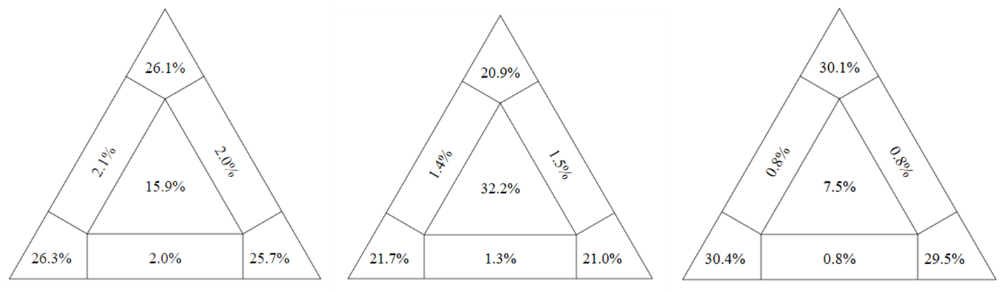


RVA-G6

RVA-G6(IV)

RVA-G6(III)

**2) Absence of nucleotide substitution saturation:**

Substitution saturation in an alignment is the result on multiple substitution at the same site in a sequence, or identical substitution in different sequences (Philippe et al., 2011). When this happens, the apparent distances underestimate the real genetic distance, decreasing the phylogenetic information contained in the sequences (Xia et al., 2003; Philippe et al., 2011).

In the present work, the presence of substitution saturation was evaluated by the Xia´s test (Xia et al., 2003) implemented in the DAMBE software v7.2.102 (Xia, 2018). Presence of saturation was not observed in the analyzed datasets (see the following table).

| **Dataset** | **N sequences** | **I_ss_^a^** | **I_ss.c_Sym^b^** | **p-value** | **I_ss.c_Asym^c^** | **p-value** |
| --- | --- | --- | --- | --- | --- | --- |
| RVA-G6 | 4 | 0.266 | 0.820 | 0.0000 | 0.788 | 0.0000 |
|  | 8 | 0.255 | 0.787 | 0.0000 | 0.681 | 0.0000 |
|  | 16 | 0.269 | 0.770 | 0.0000 | 0.572 | 0.0000 |
|  | 32 | 0.295 | 0.746 | 0.0000 | 0.44 | 0.0000 |
| RVA-G6(III) | 4 | 0.044 | 0.81 | 0.0000 | 0.779 | 0.0000 |
|  | 8 | 0.045 | 0.773 | 0.0000 | 0.664 | 0.0000 |
|  | 16 | 0.047 | 0.754 | 0.0000 | 0.547 | 0.0000 |
|  | 32 | 0.048 | 0.728 | 0.0000 | 0.406 | 0.0000 |
| RVA-G6(IV) | 4 | 0.095 | 0.808 | 0.0000 | 0.776 | 0.0000 |
|  | 8 | 0.092 | 0.769 | 0.0000 | 0.66 | 0.0000 |
|  | 16 | 0.09 | 0.749 | 0.0000 | 0.541 | 0.0000 |
|  | 32 | 0.091 | 0.723 | 0.0000 | 0.399 | 0.0000 |
| ^a^ Index of substitution saturation.  ^b^ Critical index of substitution saturation assuming a symmetrical topology.  ^c^ Critical index of substitution saturation assuming an asymmetrical topology.  p-value for two-tailed test. | | | | | | |

Interpretation of results:

Significant Difference

----------------------

Yes No

-------------------------------------------------------

Iss < Iss.c Little Substantial

saturation saturation

-------------------------------------------------------

Iss > Iss.c Useless Very poor

sequences for phylogenetics

-------------------------------------------------------

**3) Absence of potential recombinant strains:**

Recombination violates the basic assumption of phylogeny inference (ancestry from a common ancestor) and can bias the accuracy of the constructed phylogenetic trees, molecular clock, and coalescent estimates (Posada and Crandall, 2002). Therefore, recombinant sequences should be excluded from the analysis or should be analyzed separately with more complex coalescent models (Martin et al., 2015).

Evidence of recombination in the datasets was evaluated using the methods implemented in the RDP v4.100 software (Martin et al., 2015) with the default settings. Only recombination events with p ≤ 0.05 detected by at least four methods were considered. No evidence of possible recombination was found in the analyzed datasets.

**4) Presence of temporal signal:**

Before to infer a time-scaled tree using a molecular clock, it is important to confirm that the dataset contain sufficient temporal signal. This means there must be sufficient genetic change between sampling times to reconstruct the relationship between genetic divergence and time (Rambaut et al., 2016).

The presence of temporal structure was evaluated by regression of divergence (root-to-tip genetic distance) vs sampling time using TempEst v1.5.3 (Rambaut et al., 2016). The positive linear trend observed in the three datasets indicates that the data contain a temporal signal, appropriate for follow up analyzes (see the following figure).


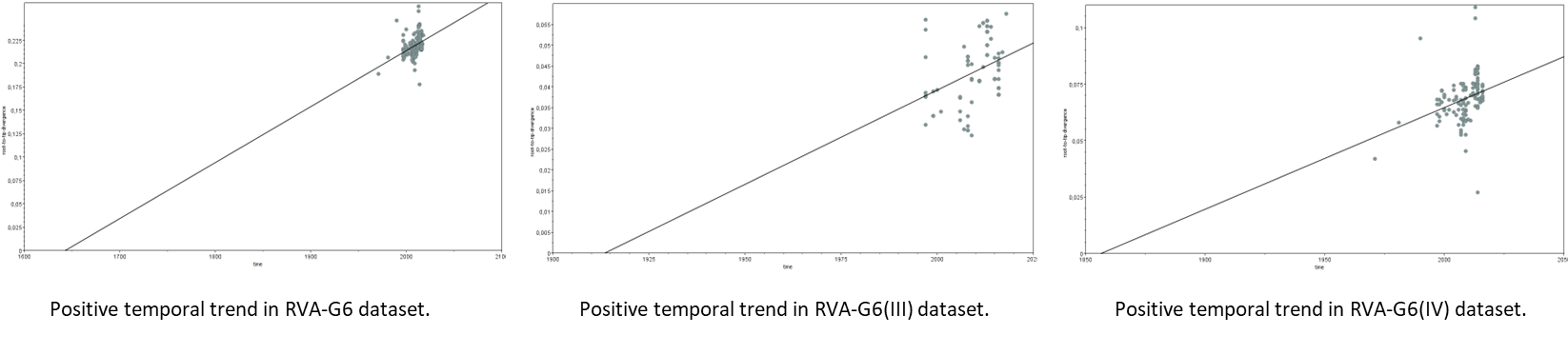


References:

Martin, D.P.; Murrell, B.; Golden, M.; Khoosal, A.; Muhire, B. RDP4: detection and analysis of recombination patterns in virus genomes. Virus Evol. **2015**, *1*: vev003-vev003. [https://doi.org/ 10.1093/ve/vev003](https://doi.org/%2010.1093/ve/vev003)

Mavian, C.; Marini, S.; Prosperi, M.; Salemi, M. A Snapshot of SARS-CoV-2 genome availability up to April 2020 and its implications: data analysis. JMIR Public. Health Surveill. **2020**, *1*: e19170. https://doi.org/[10.2196/22853](https://doi.org/10.2196%2F22853)

Philippe, H.; Brinkmann, H.; Lavrov, D.V.; Littlewood, D.T.J.; Manuel, M.; Wörheide, G.; Baurain, D. Resolving difficult phylogenetic questions: why more sequences are not enough. PLoS Biology. **2011**, *9*: e1000602. <https://doi.org/10.1371/journal.pbio.1000602>

Posada, D.; Crandall, K. The effect of recombination on the accuracy of phylogeny estimation. J. Mol. Evol. **2002**, *54*, 396-402. <https://doi.org/10.1007/s00239-001-0034-9>

Rambaut, A.; Lam, T.T.; Carvalho, L.M.; Pybus, O.G. Exploring the temporal structure of heterochronous sequences using TempEst (formerly Path-O-Gen). Virus Evol. **2016**, *2*: vew007. https://doi.org/10.1093/ve/ vew007

Schmidt, H.A.; Strimmer, K.; Vingron, M.; Von Haeseler, A. TREE-PUZZLE: maximum likelihood phylogenetic analysis using quartets and parallel computing. Bioinformatics **2002**, *18*, 502-504. <https://doi.org/10.1093/bioinformatics/18.3.502>

Strimmer, K.; Von Haeseler, A. Likelihood-mapping: a simple method to visualize phylogenetic content of a sequence alignment. Proc. Natl. Acad. Sci. **1997**, *94*: 6815-6819. <https://doi.org/10.1073/pnas.94.13.6815>

Xia, X. DAMBE7: new and improved tools for data analysis in molecular biology and evolution. Mol. Biol. Evol. **2018**, *35*, 1550-1552. https://doi.org/10.1093/molbev/ msy073

Xia, X.; Xie, Z.; Salemi, M.; Chen, L.; Wang, Y. An index of substitution saturation and its application. Mol. Phylogenet. Evol. **2003**, *26*: 1-7.<https://doi.org/10.1016/S1055-7903(02)00326-3> ”
