# Supplementary figures and images for: Molecular Epidemiology of Rotavirus A in Calves: Evolutionary Analysis of a Bovine G8P[11] Strain and Spatio-Temporal Dynamics of G6 Lineages in the Americas
Source: Viruses. 2023 Oct 19;15(10):2115. doi: 10.3390/v15102115 (PMC10611311; doi:10.3390/v15102115)

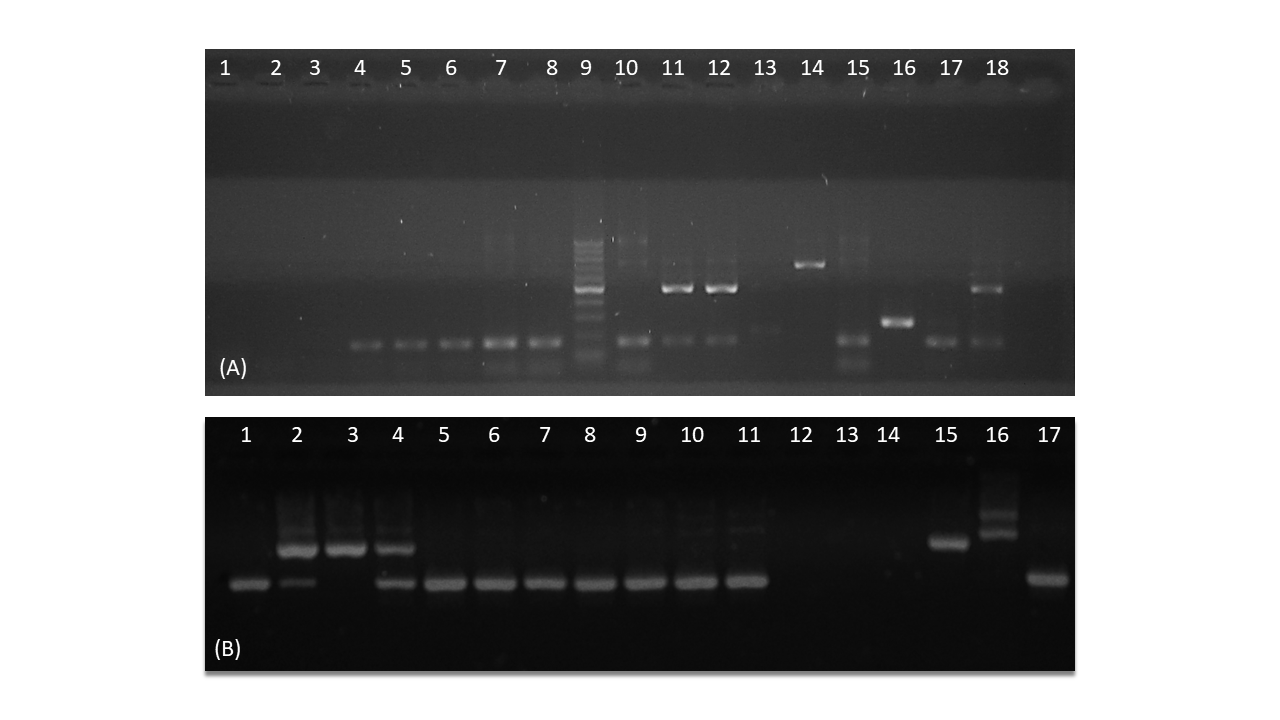

Supplement: Supplementary file 1 [file viruses-15-02115-s001.zip › SupFig S1.tiff]

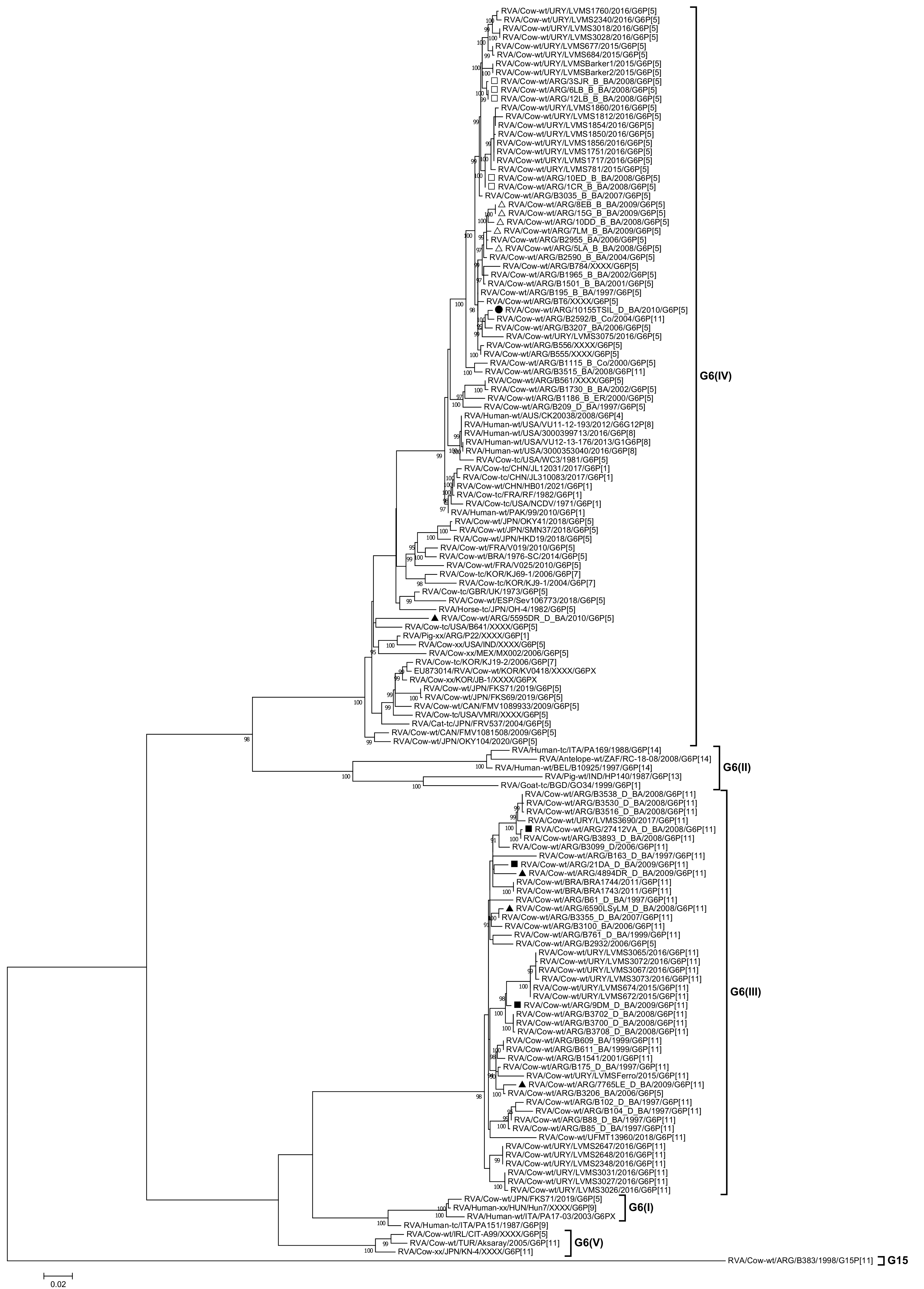

Supplement: Supplementary file 1 [file viruses-15-02115-s001.zip › SupFig S2.tiff]

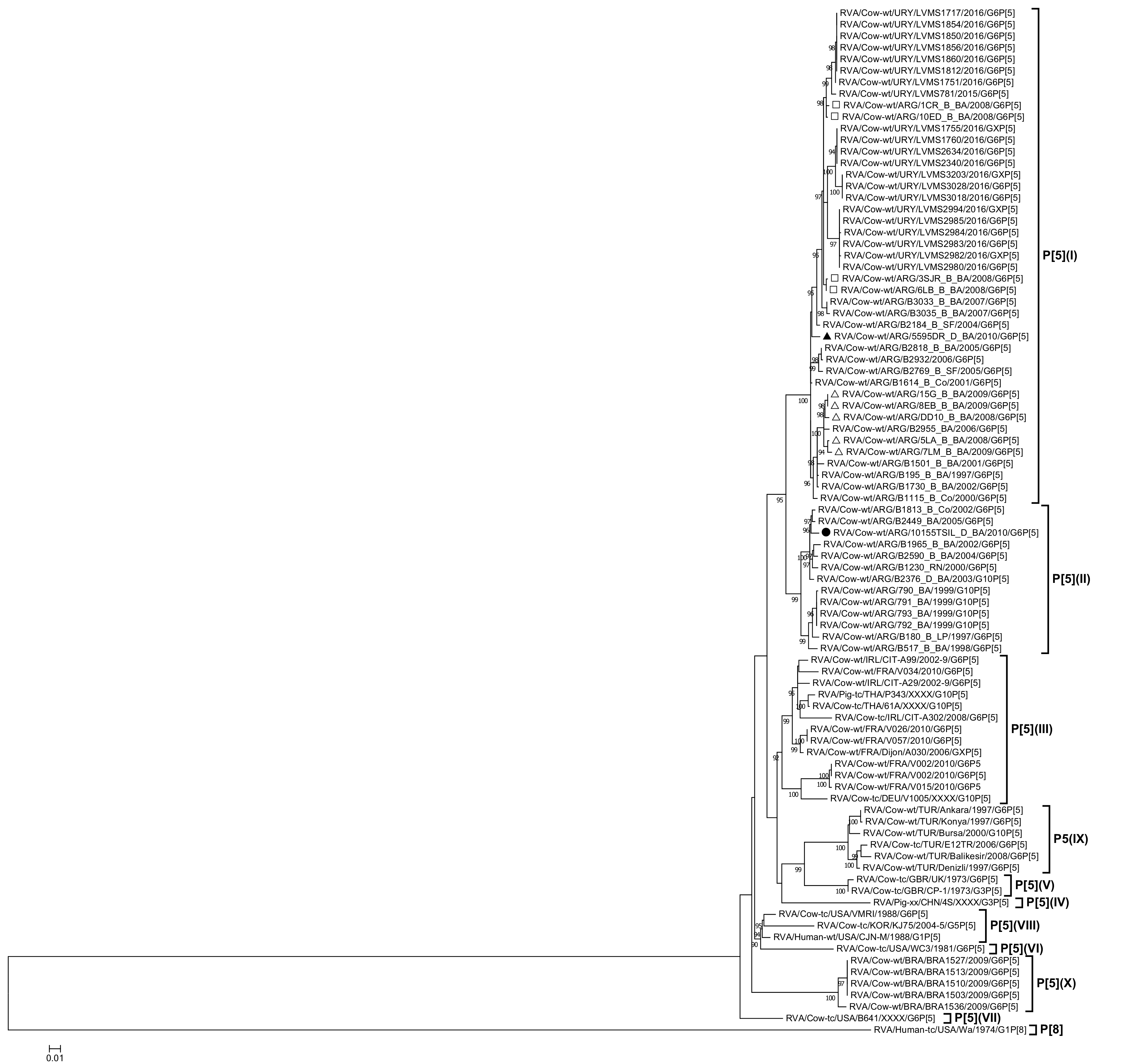

Supplement: Supplementary file 1 [file viruses-15-02115-s001.zip › SupFig S3.tiff]

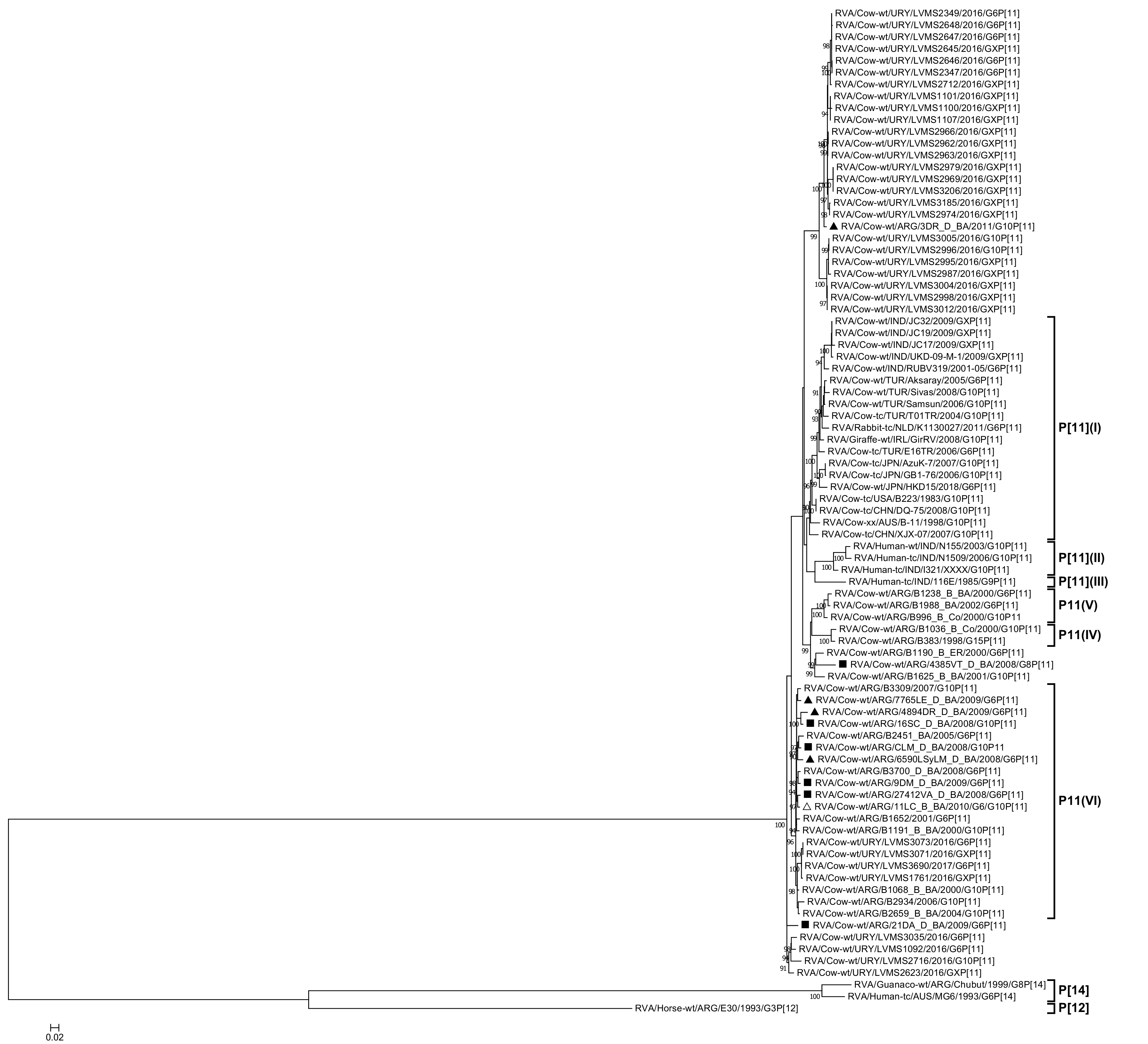

Supplement: Supplementary file 1 [file viruses-15-02115-s001.zip › SupFig S4.tiff]

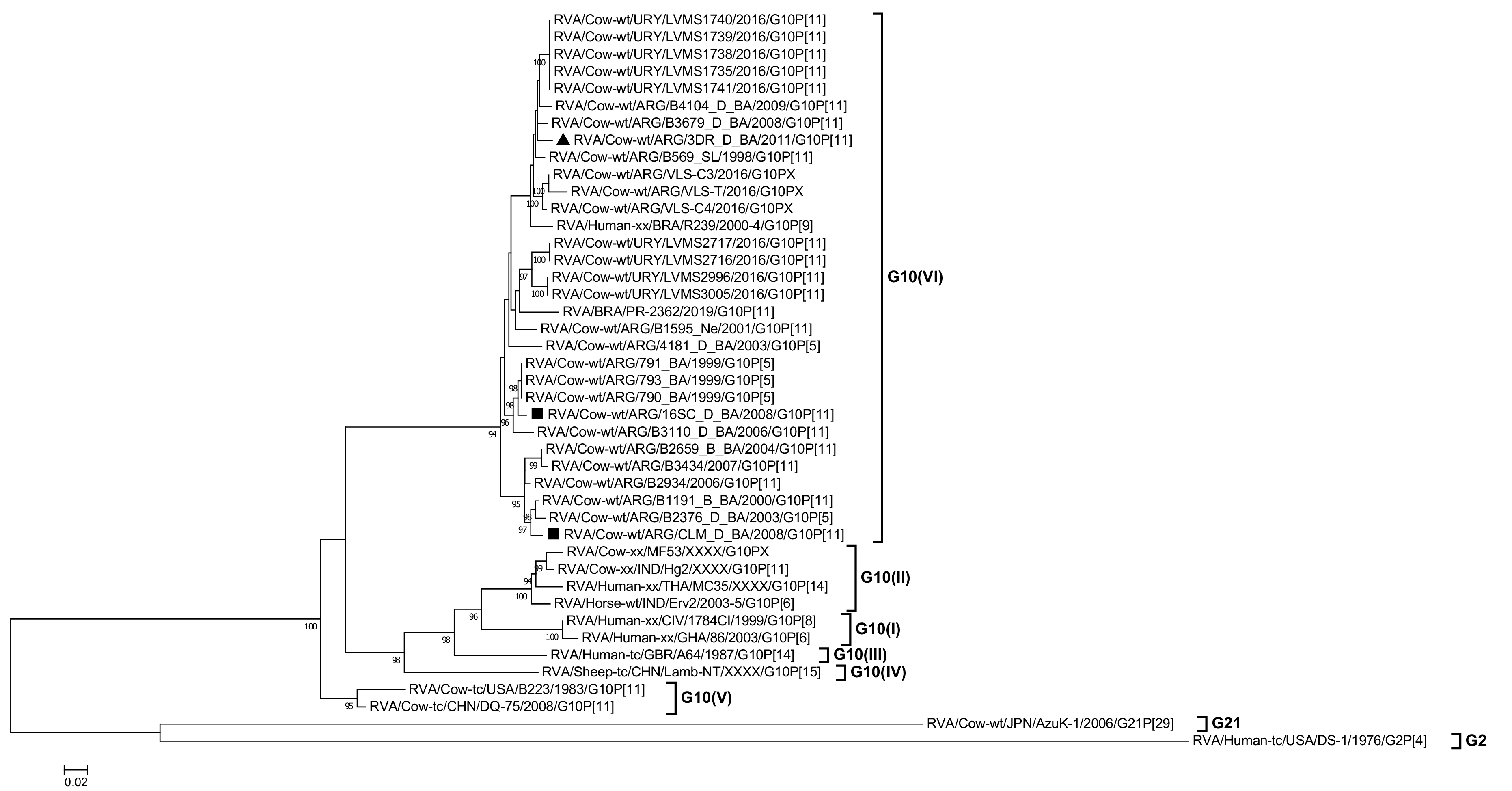

Supplement: Supplementary file 1 [file viruses-15-02115-s001.zip › SupFig S5.tiff]

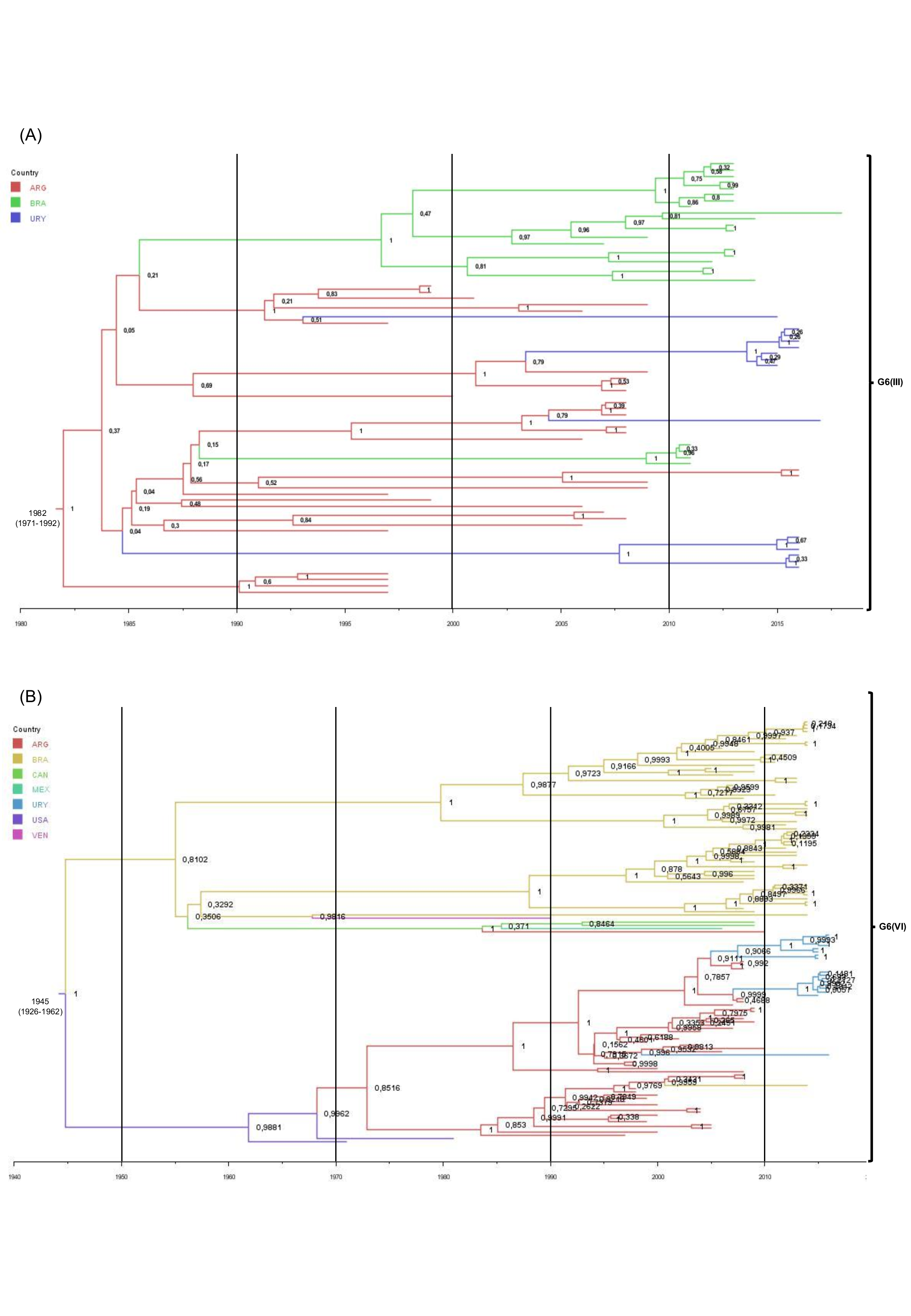

Supplement: Supplementary file 1 [file viruses-15-02115-s001.zip › SupFig S6 A and B.tiff]

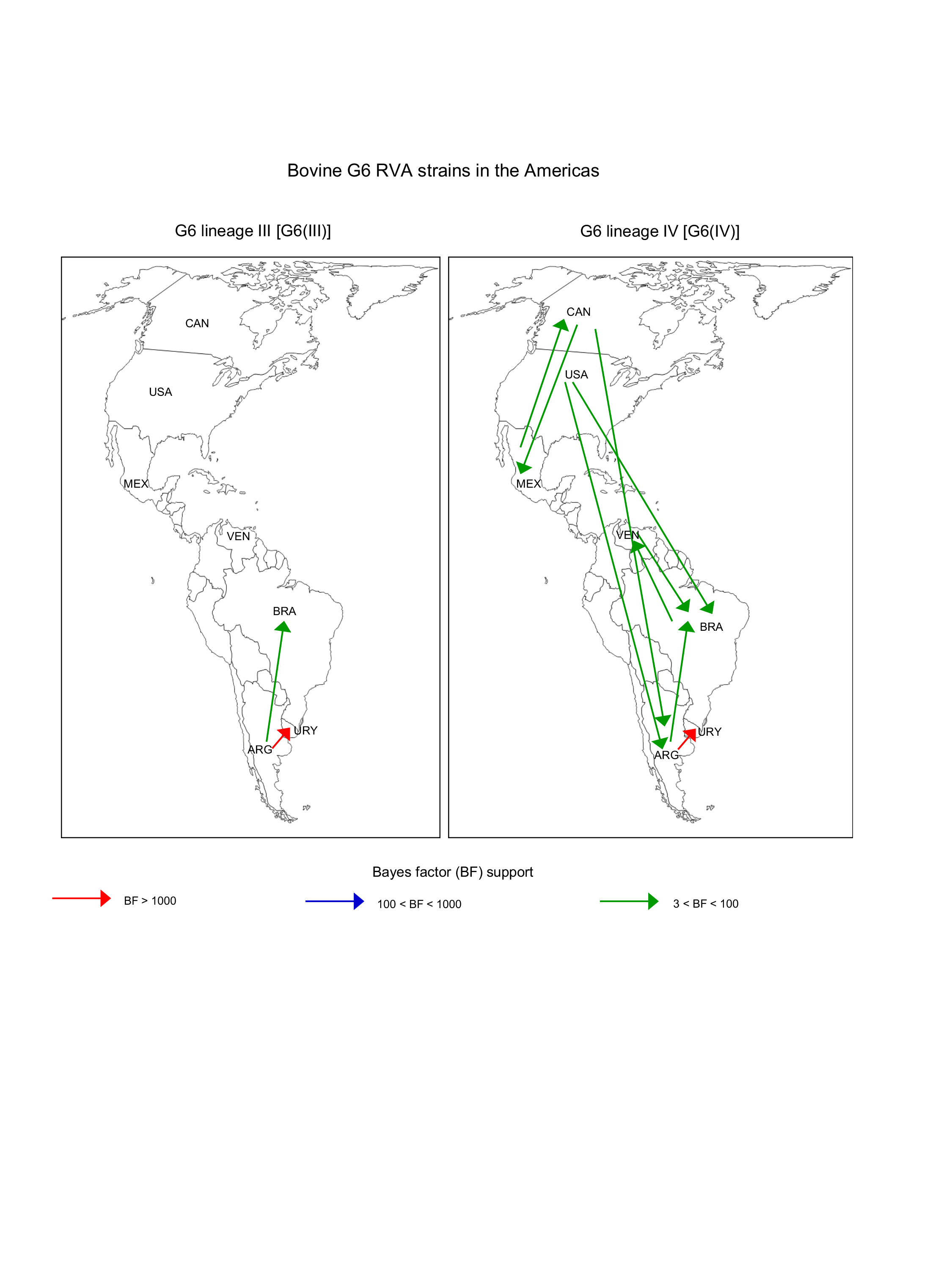

Supplement: Supplementary file 1 [file viruses-15-02115-s001.zip › SupFig S7.tiff]
